# Lipid Phosphate Phosphatases and Cancer

**DOI:** 10.3390/biom10091263

**Published:** 2020-09-02

**Authors:** Xiaoyun Tang, David N. Brindley

**Affiliations:** 1 Department of Biochemistry, University of Alberta, Edmonton, AB T6G 2S2, Canada; xtang2@ualberta.ca; 2 Cancer Research Institute of Northern Alberta, University of Alberta, Edmonton, AB T6G 2E1, Canada

**Keywords:** PAP-2, autotaxin, lysophosphatidate, G protein-coupled receptor

## Abstract

Lipid phosphate phosphatases (LPPs) are a group of three enzymes (LPP1–3) that belong to a phospholipid phosphatase (PLPP) family. The LPPs dephosphorylate a wide spectrum of bioactive lipid phosphates, among which lysophosphatidate (LPA) and sphingosine 1-phosphate (S1P) are two important extracellular signaling molecules. The LPPs are integral membrane proteins, which are localized on plasma membranes and intracellular membranes, including the endoplasmic reticulum and Golgi network. LPPs regulate signaling transduction in cancer cells and demonstrate different effects in cancer progression through the breakdown of extracellular LPA and S1P and other intracellular substrates. This review is intended to summarize an up-to-date understanding about the functions of LPPs in cancers.

## 1. Introduction

Lipid phosphate phosphatases (LPPs) consist of three enzymes (LPP1–3), which have been classified as phospholipid phosphatases (PLPP). So far, the PLPP family has seven members, PLPP1–7, in which PLPP1, PLPP2, and PLPP3 correspond to the former LPP1, LPP2, and LPP3, respectively. Mammalian LPP1–3 are encoded by three separate genes, *PLPP1*, *PLPP2*, and *PLPP3*, and they hydrolyze a wide spectrum of lipid phosphates including phosphatidate (PA), lysophosphatidate (LPA), sphingosine 1-phosphate (S1P), ceramide 1-phosphate (C1P), and diacylglycerol pyrophosphate (DGPP) in a Mg^2+^-independent and *N*-ethylmaleimide (NEM)-insensitive manner [1,2]. PLPP4 and PLPP5 are the former diacylglycerol pyrophosphate phosphatase-like 2 (DPPL2) and 1 (DPPL1), respectively. PLPP4–5 prefer DGPP as a substrate and also hydrolyze PA and LPA [3]. The activities of PLPP4–5 are also Mg^2+^-independent, but they can be inhibited by NEM [3]. PLPP6 is formerly known as polyisoprenyl diphosphate phosphatase 1 (PDP1) or candidate sphingomyelin synthase type 2β (CSS2β), which hydrolyzes presqualene diphosphate (PSDP), farnesyl diphosphate (FDP), S1P, LPA, and PA, but it has a preference for PSDP [4,5]. LPPs (PLPP1–3) and PLPP4–6 share highly conserved catalytic domains but show different substrate preferences. LPPs are responsible for the breakdown of extracellular LPA and S1P, which are two important signal molecules and therefore participate in many physiological and pathological processes such as vascular development [6], cell cycle regulation [7], cardiovascular disease [8], and cancer [9]. So far, there are very few reports about PLPP4–6, and their physiological functions are not clear. PLPP7, formerly known as NET39 or CSS2α, is catalytically inactive as a phosphatase due to the loss of critical amino acids in the catalytic domains [10,11].

The process of identifying LPPs dates back to the 1950s when a phosphatidate phosphatase (PAP) activity that dephosphorylates PA to form diacylglycerol (DAG) was discovered in mammalian tissue [12,13]. The PAP activity was intensively investigated as a critical regulator of lipid metabolism because the transformation from PA to DAG represents an intermediate reaction in the Kennedy pathway [14]. Early studies found that the cytosolic and membrane-bound PAPs exhibit quite different enzymological characteristics. For instance, the activity of the cytosolic PAP (PAP-1) that translocates onto membranes of the endoplasmic reticulum (ER) depends on the presence of Mg^2+^ and is sensitive to NEM [15,16,17,18]. Its activity is required for the synthesis of triacylglycerol, phosphatidylcholine, and phosphatidylethanolamine [19,20]. It was not until 2006 that PAP-1 was identified in yeast and was found to be the orthologue of a family of three mammalian proteins called lipins [21]. Then, all three of the mammalian lipins were shown to have PAP activity, which is involved in glycerolipid synthesis [22].

A Mg^2+^-independent phosphatidate phosphatase activity (PAP-2) was also described, and this activity was found mainly in the plasma membrane fraction [23]. This activity in mammals was not inhibited by NEM, which further distinguished it from PAP-1 activity. This new class of PAP activities was characterized in liver [23,24,25,26]. Unlike PAP-1, which is specific for PA, PAP-2 degrades a wide spectrum of phospholipids including PA, LPA, S1P, C1P, and lipid pyrophosphates in vitro [1]. This observation led to the more accurate naming of the PAP-2 activity as a lipid phosphate phosphatases [27]. The identification of PAP-2 at a molecular level was achieved by the revelation of cDNA sequences of three PAP-2 isoforms (PAP-2a, PAP-2b, and PAP-2c) in human beings and other animals [28,29,30,31]. These isoforms share amino acid sequence homology, and LPP orthologs were also identified in fruit flies and yeast [32,33,34].

mRNA of LPP1–3 are universally expressed in different tissues of human beings including adrenal, appendix, bone marrow, brain, colon, duodenum, endometrium, esophagus, fat, gall bladder, heart, kidney, liver, lung, lymph node, ovary, pancreas, placenta, prostate, salivary gland, skin, small intestine, spleen, stomach, testis, thyroid, and urinary bladder [35]. Protein expression data from The Human Protein Atlas (http://www.proteinatlas.org) indicate that LPP1–3 are expressed in most tissues, among which LPP1 is highly expressed in the prostate and kidney. LPP2 is expressed at a higher level in the gastrointestinal tract, salivary gland, gallbladder, pancreas, kidney, urinary bladder, and brain than in other tissue, while LPP3 is high in lung, salivary gland, oral mucosa, duodenum, smooth muscle, and skin [36].

Bioactive phospholipids such as LPA and S1P in the extracellular environment signal through their families of G protein-coupled receptors to induce a plethora of effects including cell survival, migration, vascular formation, and inflammation, which play critical roles in cancer development. Functioning as integral membrane phospholipid phosphatases, LPPs hydrolyze extracellular LPA/S1P and attenuate their downstream signaling. LPPs are also present in the intracellular membranes such as the ER and Golgi network [37]. This allows LPPs to hydrolyze intracellular lipid phosphates that have access to the active sites of the LPPs, and thus, the LPPs affect intracellular signaling pathways. Considerable evidence has been accumulated about the functions of LPPs (PLPP1–3) in many physio-pathological processes, including cancer. This review is intended to summarize an up-to-date understanding of the roles of LPPs in cancer development and offer insights for the future directions of cancer treatment.

## 2. Structure and Membrane Topology of LPP

**Figure 1:**
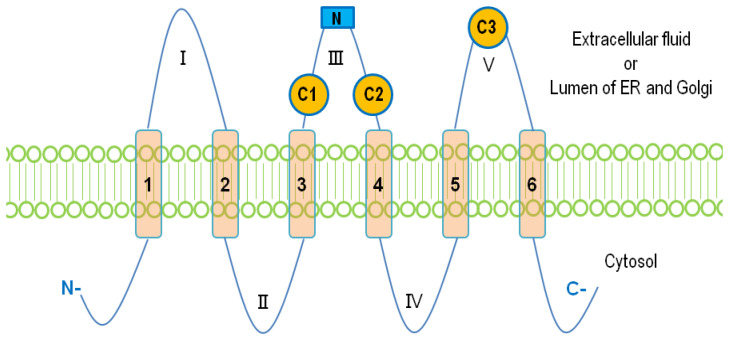
The membrane topology of lipid phosphate phosphatases (LPPs). Six membrane-spanning regions (1–6) are connected with five extramembrane loops (I–V). Three conserved catalytic domains, C1, C2, and C3, are located on loops III and V. The N-linked glycosylation site on the loop III is shown as a blue square.

**Figure 2:**
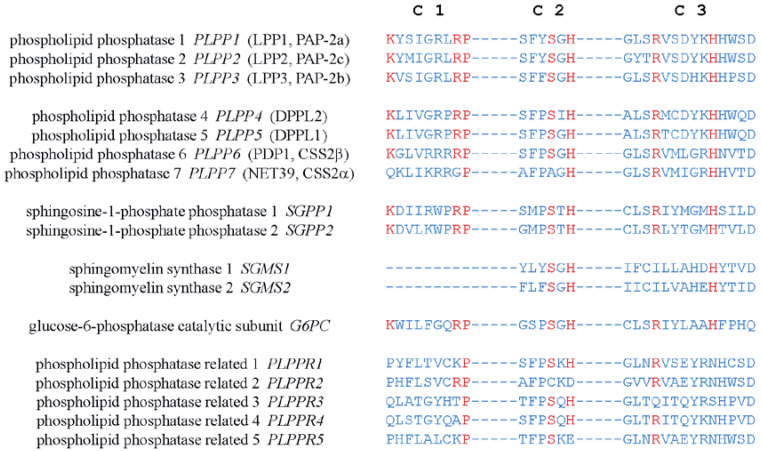
Amino acid sequences of the conserved catalytic domains, C1, C2, and C3, in human LPPs and other proteins with structure similarity. Residues critical for the catalytic activity are shown in red.

The mammalian LPPs are localized on the plasma membrane and intracellular network of ER and Golgi [7,37]. It has been reported that LPP1 and LPP3 are present in lipid rafts or caveolae [38,39]. There is also evidence that LPP1 can be directed to the apical surface membrane by a FDKTRL motif on the N-terminus, whereas LPP3 is accumulated at the basolateral membrane [40]. The crystal structure of the LPPs has not yet been solved. A putative topology for the LPPs was determined based on the data obtained from hydrophobicity plots and transmembrane disposition analysis of the rat Dri42 protein [41], which later proved to be rat LPP3 [28]. It has six membrane-spanning regions connected by five extramembrane loops (I–V). Both C- and N-terminal extensions and loop II and IV are located in the cytosol. Loops I, III, and V are on the extracellular side of the membrane. (Figure 1). Three conserved domains (C1, C2, and C3) that form the catalytic site are located on loops III and V outside the cells. Residues that are indispensable for the phosphatase activity in C1–C3 (Figure 2) were identified by amino acid substitution analysis [42]. LPPs inside the cells are localized in the ER [37,41] and Golgi [28]. There is an N-linked glycosylation site between C1 and C2 (Figure 1) [42], indicating that the catalytic site are on the luminal side of ER and Golgi where LPPs are glycosylated [42]. This topology enables LPPs to hydrolyze substrates outside of the cells and in the lumen of ER and Golgi [9,43].

The catalytic mechanism of LPPs has been postulated and proposed through a combination of computational modeling and the crystal structure of chloroperoxidase, which is a related enzyme that also possesses the C1–3 domains [44,45]. The conserved histidine on C3 serves as the nucleophile acting on the phosphate group to form a phospho-histidine intermediate. The C2 histidine is involved in breaking the phosphate bond. The conserved lysine and arginine on C1 as well as the arginine on C3 help coordinate the substrate in the active site [43,44,45]. Similar domains are also found in PLPP4–7. Unlike PLPP1–3, PLPP6 only has four transmembrane helices, and C1–3 of PLPP6 are located at the cytosolic side of the membrane. This allows PLPP6 to hydrolyze polyisoprenoid diphosphates in the cytosol [46]. Sphingosine phosphate phosphatases (SPPs), sphingomyelin synthases (SMSs), phospholipid phosphatase-related proteins (PLPPRs) [43,47], glucose 6-phosphatase (G6P), and *E. coli* phosphatidylglycerol-phosphate phosphatase B (PGPB), an orthologue of human G6P [33], also have the conserved catalytic domains. It is notable that the putative structure of PGPB was established through its crystal structure, which was determined later [47]. The structure of LPPs is thought to be modeled accurately from that proposed for PGPB.

## 3. Ecto-Activity of LPPs

A major part of circulating LPA is generated from lysophosphatidylcholine (LPC) through the lysophospholipase D activity of autotaxin (ATX) [48,49]. LPC is abundant in circulation with a concentration (>200 μM in human beings) [50], which is much higher than the K_m_ of ATX for LPC (approximately 100 μM) [51]. As a secretary enzyme, ATX can readily access the LPC pool to generate LPA.

S1P is a sphingolipid analogue of LPA. The precursor for S1P synthesis is sphingosine, which is formed through the hydrolysis of ceramide by ceramidases. Sphingosine is phosphorylated by sphingosine kinase-1 and -2 (SPHK1 and 2) inside cells to generate S1P. SPHK1 is cytosolic and it interacts with the plasma membrane, whereas SPHK2 is present in the mitochondria [52] and nuclei [53]. S1P can be exported out the cells by the membrane transporters including ATP-binding cassette (ABC) transporters (ABCC1, ABCG2, and ABCA1) [54,55,56,57], spinster homolog-2 (SPNS2) [58], and major facilitator superfamily transporter 2b (Mfsd2b) [59]. This facilitates the “inside-out signaling” of S1P [54].

Both LPA and S1P outside the cells induce a plethora of cellular responses such as proliferation, migration, angiogenesis, and inflammation [51,60,61,62] through receptors on the cell surface. To date, six LPA receptors (LPAR1–6) and five S1P receptors (S1PR1–5) have been identified and all of them are G protein-coupled receptors (GPCRs). Plasma membrane-localized LPPs dephosphorylate extracellular LPA and S1P and thereby attenuate LPA/S1P signaling.

The ecto-phosphatase activity was established in rat2 fibroblasts where the overexpression of LPP1 increased the dephosphorylation of extracellular LPA, PA, and C1P. This action attenuated LPA-induced MAPK (mitogen-activated protein kinase) activation and inhibited cell migration [37,63,64]. Similarly, LPP1 and LPP2 inhibited the activation of MAPK that was stimulated by LPA or S1P in HEK293 cells [65]. The dephosphorylation of LPA generates monoacylglycerol (MAG), which can be transported into the cells and re-phosphorylated to form intracellular LPA [66]. This intracellular LPA can activate LPA1 receptors on the nuclear membrane and stimulate the expression of cyclooxygenase-2 (COX-2) and inducible nitric oxide synthase (iNOS) [67]. Intracellular LPA has also been reported to initiate signaling through peroxisome proliferator-activated receptor γ (PPARγ) [68].

The ecto-activity of LPPs in vivo is more complex. Exogenous LPA injected into the circulation is turned over rapidly with the half-life of approximately 1 min [69]. LPP1 knockout mice showed increased levels and a decreased turnover rate of circulating LPA [70]. A similar phenotype was observed in LPP1 hypomorph mice, which have a low expression of LPP1 in most organs except the brain [71]. Interestingly, mice that transgenically overexpressed LPP1 did not show a decrease in the circulating LPA concentrations [72], suggesting that other factors may affect the ecto-activity of LPPs in vivo. For instance, the activity of LPPs is inhibited strongly by Ca^2+^, which is present in the extracellular environment at approximately 2 mM [37]. In addition, the physiological concentration of LPA in the plasma (0.1–1 µM) is much lower than the Km of LPP1 for LPA (approximately 36 μM) [37]. This indicates that the ecto-activity of LPPs is more important when the LPA levels are increased. In cancers, extracellular LPA levels are elevated as high as 10 μM [73,74,75]. We do not know if LPA concentrations in the vicinity of the LPPs are modified by other factors such as the levels of expression of the LPA receptors.

S1P concentrations in the plasma range from 100 nM to 1 μM [54]. Exogenous S1P injected into the circulation is cleared from the blood in 15–30 min [76]. S1P is dephosphorylated by SPPs and LPPs, or irreversibly cleaved by S1P lyase (SPL). SPPs and SPL are localized on the ER [77,78]. Therefore, the plasma membrane-localized LPPs have an essential role in regulating the amount of extracellular S1P. The ecto-activity of LPP1 and LPP3 against S1P has been demonstrated in cells [69,79] and animals [80,81]. LPP3 and LPP1a, a splice variant of LPP1, seem to be more efficient at hydrolyzing S1P than LPP1 and LPP2. Phospho-FTY720, an analogue of S1P, can be converted to FTY720 by LPP3 and LPP1a [82], but not by LPP1 or LPP2 [83]. Similarly, expressing exogenous LPP1, LPP2, or LPP3 in HEK293 cells enhances the ecto-activity against LPA, but only LPP3 significantly increases the degradation of extracellular S1P [69]. Sphingosine formed by the dephosphorylation of S1P can be transported into the cells and re-phosphorylated into S1P [79]. Therefore, this process represents a mechanism for the entry of S1P into cells.

## 4. Intracellular Activities of the LPPs

Not all of the effects of LPPs can be attributed to their ecto-activities. LPP1 is able to suppress wls-31-induced cell migration and Ca^2+^ mobilization [64,84]. Wls-31 is an isosteric phosphonate analog of LPA that activates LPAR1/2, but cannot be hydrolyzed by LPPs. LPP1 and LPP2 also inhibit MAPK activation induced by thrombin, which activates protease-activated receptors (PARs) [65]. Similarly, Ca^2+^ mobilization induced by a PAR1 peptide in MDA-MB-231 cells is inhibited by increased LPP1 expression [84]. Furthermore, LPP1 decreases the platelet derived growth factor (PDGF)-induced migration of embryonic fibroblasts through inhibiting the PDGF/PKC (protein kinase C) /MAPK pathway [63]. These effects of the LPPs are independent of their ecto-activities because these agonists cannot be degraded by LPPs. However, the effect requires LPP activity and therefore probably depends on the degradation of an intracellular lipid phosphate that is formed downstream of the activation of LPA, PAR, or PDGF receptors.

LPPs are also present on the ER and Golgi network with the catalytic domains, which should face the luminal side. As such, LPPs probably have specific access to substrates depending on the subcellular compartment. One of these possible substrates inside the cells is PA, which activates Sos (son of sevenless), Raf (rapidly accelerated fibrosarcoma), MAPK, mTOR (mammalian target of rapamycin), AKT (Ak strain transforming), and SPHK1 [85,86,87]. The dephosphorylation of PA generates DAG, which activates the classical and novel PKCs and Ras (rat sarcoma) guanyl nucleotide-releasing protein [88]. Increasing LPP1, LPP2, or LPP3 does decrease intracellular PA/DAG ratios [38,89]. LPP3 depletion decreases the levels of de novo synthesized DAG and the Golgi-associated DAG [90]. LPP2 decreases intracellular PA, which promotes the apoptosis of HEK293 cells in serum-deprived media [91]. However, LPP3 or LPP1 did not change intracellular DAG significantly in other studies [65,72,92].

Since the catalytic domains of LPPs are on the luminal side of ER and Golgi or the outer surface of the plasma membrane, the LPPs should not be able to dephosphorylate PA, which is formed at the cytosolic side of the membranes, unless the PA can be transported across the membranes to the catalytic sites of LPPs. However, this has yet to be shown. It should be noted that increasing LPP1 activity directly inhibits phospholipase D (PLD) activation [64], which forms a large proportion of intracellular PA. This can provide an alternative explanation for the decreased accumulation of PA. It is likely that the lipins, which are cytosolic phosphatidate phosphatases that translocate to membranes, are responsible for the degradation of most of the PA on the cytosolic surface of membranes [93].

LPPs probably also degrade intracellular C1P and S1P, both of which are involved in inflammation. C1P activates phospholipase A_2_ (PLA_2_) to produce arachidonate, which is converted to inflammatory eicosanoids (prostaglandins and thromboxanes) by COX-1/2 [94]. S1P helps to coordinate the metabolism of arachidonate by COX-2 to ensure the maximum production of prostaglandin E2 (PGE_2_) [94]. S1P also interacts with specific intracellular target proteins such as histone deacetylase 1/2, prohibitin 2, PPARγ, and tumor necrosis factor (TNF) receptor associated factor 2, to induce cell responses [95]. The overexpression of LPP3, but not LPP2, decreases intracellular S1P in HEK293 cells [91]. The degradation of intracellular S1P can be performed by other enzymes such as S1P phosphatases and S1P lyase, which are major regulators of intracellular S1P concentrations.

## 5. Upregulation of LPA Signaling in Cancers

Functioning as a platelet activator, a chemoattractant, and a growth factor, LPA plays a critical role in wound healing [96]. At sites of tissue damage, LPA stimulates the proliferation of fibroblasts and endothelial cells [97], and it promotes collagen deposition [98] and angiogenesis [99,100]. Circulating LPA concentrations are normally between 0.1 and 1 μM [51], and this is regulated mainly by the balance of ATX activity versus that of the LPPs.

LPA signaling is magnified and hijacked by cancers (wounds that do not heal) [101]. Elevated ATX levels have been observed in the blood and malignant tissues from patients with thyroid [102], lung [103], breast [104], liver [105], pancreatic [106,107], kidney [108], bladder [108], and prostate cancer [109]. As a consequence, LPA levels increase in those cancers [107,110,111,112,113], which has been considered an indicator of poor prognosis [110,113]. Significantly, LPA concentrations have been reported to reach as high as 10 μM in the ascites fluid of ovarian cancer patients [73,74,75]. Cancer cells express high levels of LPAR1–3 [61], which are GPCRs. LPAR1–3 couple to G proteins: Gi/o, Gq/11, and G12/13 [61], and activate PI3K (phosphoinositide 3-kinase) /AKT [114,115], PLC (phospholipase C) [116], and Rho pathways [116]. LPAR1–3 are elevated in brain [117,118], pancreatic [119,120,121], colon [122,123], and breast cancer [124], which is associated with enhanced tumor growth and metastasis.

Introducing exogenous LPAR1 converts non-transformed MCF-10A cells into an invasive phenotype [125]. LPAR1 and/or LPAR3 activate Wnt/β-catenin and PI3K/AKT/mTOR pathways to induce the epithelial-to-mesenchymal transition (EMT) [126,127], which is an essential step during cancer cell stemness [128]. Cancer stem cell (CSC)-related genes such as ALDH1A1, OCT4, and SOX2 are upregulated by activating LPAR1 [129]. Blocking ATX or LPAR2 suppresses the growth of breast cancer stem cells [62,130] in which LPP3 expression is downregulated [131]. Transgenic mice overexpressing ATX or any of LPAR1–3 by MMTV-LTR (mouse mammary tumor virus long terminal repeat) promoter in mammary epithelial cells show an increased development of spontaneous breast tumors and subsequent metastases [132]. LPAR4–6 are closely related to purinergic receptors [61]. LPAR4 (P2Y9/GPR23) and LPAR5 (GPR92) in cancer cells demonstrate inhibitory effects on proliferation and migration/invasion [133,134,135,136], which is in contrast to the effects of LPAR1–3. It is notable that LPAR5 suppresses the function of infiltrated CD8+ cytotoxic T cells as a mediator of immune suppression in the tumor microenvironment (TME) [137]. The effects of LPAR6 (P2Y5) in cancers are uncertain [138,139] and require further investigation.

LPA induces lymphocyte homing [140] and the transformation of monocytes to macrophages [141], which provokes inflammation [102,142]. LPA is closely related to the inflammatory milieu in conditions such as pulmonary fibrosis, rheumatoid arthritis, atherosclerosis, and inflammatory bowel disease [143]. The TME is also characterized by chronic inflammation, which is one of the hallmarks of cancers [144]. Increasing evidence reveals that there is crosstalk between LPA signaling and cancer-related inflammation. TNFα increases ATX production by Huh7, HepG2, and Hep3B liver cancer cells through activating nuclear factor κB (NFκB). The subsequent increase in LPA enhanced the invasiveness of the cancer cells [145]. The secretion of IL-8 is increased by LPA in human bronchial epithelial cells, which is mediated by protein kinase Cδ (PKCδ) and NFκB [146,147]. IL-8 and IL-6 expressions in ovarian cancer cells are also increased by LPAR2 or LPAR3 activation [148]. In a colon cancer model, LPAR2 knockout mice formed smaller tumors after induction with azoxymethane (AOM)/dextran sulfate sodium (DSS). This was accompanied by decreased levels of COX2 and CCL2 and reduced macrophage infiltration [149]. Zhao et al. showed that LPP1 inhibits LPA-induced NFκB translocation, which blocks IL-8 secretion in human bronchial epithelial cells [150]. This suggests an important role of LPP1 in inflammation [50,142].

We recently proposed a model of the ATX–LPA inflammatory cycle in breast cancer [151,152]. In this model, tumor-derived inflammatory cytokines such as TNFα and IL-1β increase ATX secretion by the adjacent mammary adipose. As a consequence, LPA levels increase in the TME. The increased LPA stimulates cancer cells to produce more cytokines, which can overcome the LPA-mediated feedback inhibition of mRNA expression for ATX [153] to form a feed-forward inflammatory cycle. This ATX–LPA inflammatory cycle can be exacerbated by radiotherapy (RT), since irradiation increases COX-2 and inflammatory cytokines in cultured adipose tissues as well as in the fat pads of mice [154,155]. ATX and LPAR1/2 levels are also elevated by irradiation. Dexamethasone, an anti-inflammatory glucocorticoid, attenuates the RT-induced upregulation of the expression ATX and LPA1R and LPA2R and increases LPP1 expression [156], which together decrease LPA signaling. Pulmonary fibrosis caused by RT or bleomycin is also blocked by dexamethasone [157,158,159].

It is well documented that LPA signaling promotes cell survival by inhibiting the intrinsic and extrinsic apoptosis pathways [160]. LPA decreases the level of the Fas receptor and reduces the expression of the Fas ligand [161,162], which makes cancer cells less responsive to the extrinsic pro-apoptotic stimuli. LPA also attenuates the intrinsic apoptosis pathway by increasing Bcl-2 and inhibiting Bad and Bax [163,164]. These effects of LPA depend on the activation of the PI3K–Akt pathway. The decrease in the sensitivity of cancer cells to chemotherapy and RT is contributed, at least partly, by the upregulation of LPA signaling. LPA decreases the effectiveness of Taxol [165], tamoxifen [166], and doxorubicin [167] in killing breast cancer cells.

**Figure 3:**
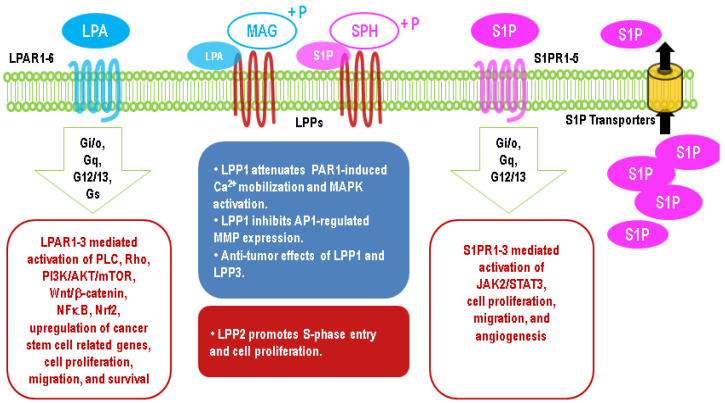
Major effects of upregulation of lysophosphatidate (LPA) and sphingosine 1-phosphate (S1P) signaling in cancer cells through G protein-coupled receptors and different functions of LPP1/3 and LPP2 in cancers.

The critical role of LPAR2 in protecting cells from radiation-induced damage has been illustrated by LPAR2 knockout mice, which exhibit increased irradiation-induced apoptosis in intestinal tissue [168]. By contrast, the knockout of LPAR1 or LPAR3 does not have this effect [168]. On the other hand, LPAR2 agonists show a therapeutic potential against irradiation-induced injury [168,169]. LPA contributes to the resistance of 786-O renal cancer cells to Temsirolimus and Sunitinib by activating Arf6 GTPase through LPAR2 [170]. Similarly, blocking LPAR1/3 with Ki16425 in resistant UMRC3 renal cancer cells re-establishes the sensitivity to Sunitinib [171]. The long-term culture of PANC-1 pancreatic cancer cells in the presence of cisplatin results in an upregulation of LPAR3 [172]. LPA through the activation of LPA1R and PI3K stabilized the expression of nuclear factor erythroid 2-related factor 2 (Nrf2), a transcription factor, which through the anti-oxidant response element increases the expression of the multidrug-resistant transporters, anti-oxidant genes, and enzymes of DNA repair [166,167,173,174]. Thus, the ATX inhibitors, ONO-8430506 and GLPG1690, enhance the sensitivity of breast tumor to doxorubicin and RT [167,175]. It should be noted that the later effect of GLPG1690 involved decreased cell division in the cancer cells, and this is compatible with the major effect of RT in solid tumors being to increase cell senescence rather than apoptosis [176,177,178]. Effects of the upregulation of LPA signaling in cancer cells are summarized in Figure 3.

## 6. Upregulation of S1P Signaling in Cancers

Elevated expression of SPHK, especially SPHK1, has been well documented in multiple cancers where the consequent increase in S1P promotes cell survival, growth, and invasiveness [179,180,181]. The overexpression of wild-type SPHK1, but not the inactive mutant, transforms NIH3T3 cells into fibrosarcoma [182]. The function of SPHK2 in cancer is unclear. Some studies indicated that SPHK2 has an opposite role to SPHK1; for instance, SPHK2 induces cell cycle arrest and promotes apoptosis [183,184,185]. The knockdown of SPHK2 enhances apoptosis and sensitivity to chemotherapy in lung and colon cancer cells [186,187]. However, emerging evidence has revealed the anti-tumor effect of SPHK2. Targeting SPHK2 demonstrates antitumorigenic effects in cancer cell lines and mouse models [186,188,189,190]. Neubauer et al. recently reported that the effect of SPHK2 on cancer depends on its expression level [191]. Moderate increases in SPHK2 promoted cell proliferation and survival, and this can be suppressed by highly overexpressed SPHK2. Interestingly, this study indicated that the highly overexpressed SPHK2 is accumulated in the nuclei, whereas at lower levels of expression, SPHK2 is on the plasma membrane. This suggests the importance of localization for the effect of SPHK2. Indeed, elevated SPHK2 has been shown in bladder, melanoma, esophageal, breast, lymphoma cancers, and leukemia [191], and this is linked to a poor prognosis in non-small cell lung cancer [192]. S1P concentrations increase in mouse and human breast tumors and in the serum of stage III breast cancer patients [193,194].

S1PRs are GPCRs. S1PR1 couples to Gi/o. It has an essential role in activating JAK2 (janus kinase 2), which causes a persistent STAT3 (signal transducer and activator of transcription 3) activation in cancers. The activated STAT3 increases the expression of S1PR1 further to form a feed-forward loop of S1PR1–JAK2–STAT3 [195]. This feed-forward loop drives tumorigenesis and metastasis [196] and contributes to the formation of the chronic inflammation milieu in colon cancer [197]. Enhanced S1PR1/STAT3 signaling has also been found in intestinal and lung cancers [197,198,199]. S1PR1 is required for tumor angiogenesis [200]. S1PR2 and S1PR3 couple to Gi/o, Gq/11, and G12/13. Functioning as a promoter of tumorigenesis and angiogenesis [201,202,203], S1PR3 is upregulated in lung cancer cells [204], and it is the most highly expressed S1PR in breast cancer cells [205]. The function of S1PR2 in cancer is uncertain, because both positive and negative impacts of S1PR2 were reported by different studies [206,207]. Compared with S1PR1–3, S1PR4 and S1PR5 have a restricted distribution and less clear functions. Effects of the upregulation of S1P signaling in cancer cells are summarized in Figure 3.

## 7. Alterations of LPP Expression in Cancers

The downregulation of LPPs results in an exacerbation of the excessive LPA and S1P signaling in cancers. LPP1 and LPP3 levels are significantly decreased in colon and breast tumors compared with the normal tissue [208,209]. Microarray data also demonstrated the downregulation of LPP1 or LPP3 in many other cancers [210,211,212]. We compared mRNA levels of LPP1–3 in all the tumors versus normal datasets of the Oncomine database, with the following threshold settings: *p* value, 0.05; fold change, 2; gene rank, top 10%. The results showed that LPP1 expression is significantly downregulated in melanoma, sarcoma, leukemia, bladder, breast, colorectal, kidney, lung, and ovarian cancers, and it is upregulated in lymphoma, brain and central nervous system, and prostate cancers. LPP3 is downregulated in melanoma, myeloma, sarcoma, bladder, breast, cervical, colorectal, lung, kidney, liver, and head and neck cancers, and it is upregulated in lymphoma.

**Figure 4:**
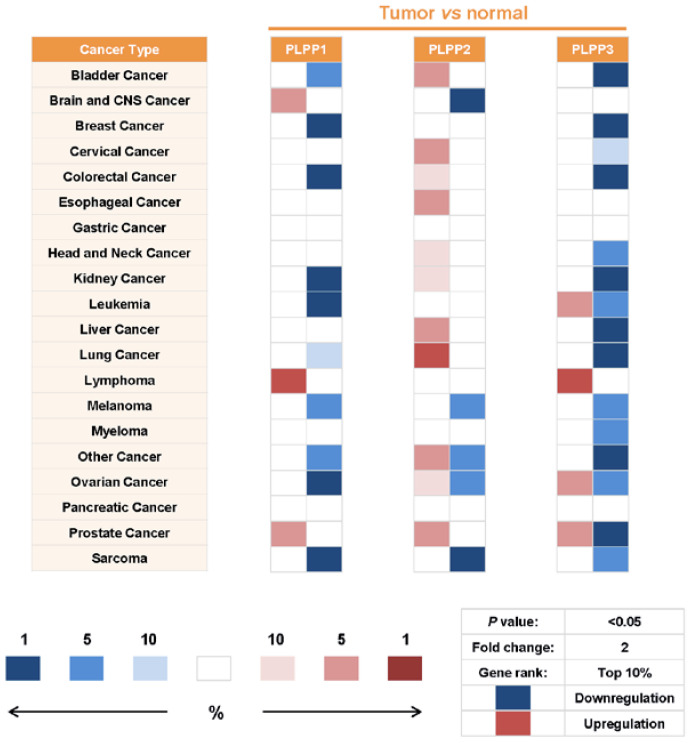
Alterations of mRNA levels of LPP1–3 in different tumors versus normal tissues. Values were obtained from the Oncomine database. The searching thresholds were set as follows: *p*-value, 0.05; fold change, 2; gene rank, top 10% (means 9% other genes have more significant *p*-values). The red or blue color represents the up- or downregulation of genes respectively in tumors relative to the adjacent normal tissue. The darkness of color corresponds to the gene rank; darker color means higher rank. LPP1 (PLPP1) and LPP3 (PLPP3) are downregulated, whereas LPP2 (PLPP2) is upregulated in the majority of cancers (*p* < 0.05). In some cases, such as PLPP2 in ovarian cancer and other cancers and PLPP3 in leukemia, ovarian cancer, and prostate cancer, both upregulation and downregulation are shown by different datasets. These cases are considered as neither upregulation nor downregulation.

In contrast, LPP2 is upregulated in 9 out of 20 categories of cancers including bladder, cervical, colorectal, esophageal, head and neck, liver, and prostate cancers, and it is downregulated in brain and central nervous system cancer, melanoma, and sarcoma (Figure 4).

Alterations (amplification, deletion, and mutation) of *PLPP1–3* are not common in cancers. How the expression of LPPs is regulated remains unclear so far. Several transcription factors that control the expression of LPPs have been identified. LPP1 can be induced by DAF-16, an orthologue of FOXO (class O forkhead box protein) transcription factors in *Ancylostoma caninum* [213]. The conditional knockout of SP2 in the mouse cerebral cortex leads to a decrease in LPP1 expression [214]. Oxidized low-density lipoprotein increases LPP3 expression in human macrophages through transcription factor C/EBPβ (CCAAT-enhancer-binding protein β) [215]. LPP3 expression can also be activated by NFκB through three response elements in the promoter region of *PLPP3* [216]. DNA modification is another mechanism changing the expression of LPPs. DNA methyltransferase Dnmt3a1 upregulates LPP3 transcription in mouse embryonic stem cells [217]. In addition, LPP3 expression can be elevated by androgens, EGF (epidermal growth factor), FGF (fibroblast growth factor), and VEGF (vascular endothelial growth factor) at the transcription level [28,218,219].

The ecto-activity of LPP in ovarian cancer cells can be increased by gonadotropin-releasing hormone (GnRH) [220]. So far, little is known about how the discrepant expression between LPP1/3 and LPP2 in cancers happens. Dexamethasone, an anti-inflammatory glucocorticoid, increased LPP1 expression in RT-treated breast tumors and adjacent adipose [155,157] suggesting that LPP1 could decrease in response to the inflammatory milieu created by the tumor. LPP3 expression can be decreased by hypoxia in the TME, leading to an asymmetrical redistribution of ATX and LPP1 to the leading and trailing edge of cancer cells, respectively [221]. These results suggest that the intrinsic characteristics of the TME such as inflammation and hypoxia may play an essential role in the downregulation of LPP1/3.

## 8. Effects of LPPs in Cancers

Increasing the low level of LPP1 or LPP3 in cancer cells leads to an inhibition in tumor growth and metastasis, which is partly caused by the ecto-activity. Ovarian cancer cells overexpressing LPP1 or LPP3 show an increased hydrolysis of extracellular LPA, resulting in impaired colony-forming ability and enhanced apoptosis [222,223]. GnRH increases the LPP ecto-activity in GnRH receptor-positive ovarian cancer cells, and this is attenuated by GnRH antagonism [220]. This effect of GnRH is associated with its antiproliferative actions on ovarian cancer cells.

We found that tetracyclines, a class of antibiotics, increase the degradation of extracellular LPA by breast cancer cells and HEK293 cells [69]. This is thought to occur through increasing the stability of LPP proteins, leading to an elevation in LPP ecto-activity. The clearance of [^32^P]LPA from the circulation is increased from 61% to 79% at 30 s and from 75% to 85% at 60 s [69]. Doxycycline treatment delays breast tumor growth in mice and decreases LPA levels in the plasma. Furthermore, doxycycline decreases inflammation in the tumors as indicated by a decrease in 11 inflammatory cytokines/chemokines and decreased NFκB levels in the nuclei of cancer cells [224].

Overexpressing LPP1 in MDA-MB-231 breast cancer cells decreases the Ca^2+^ mobilization that is stimulated by LPA, wls-31, and a PAR1 peptide [84]. LPP1 expression decreases cell migration and also suppresses tumor growth and metastasis in both syngeneic and xenograft mouse models. The catalytically inactive mutant (R217K) of LPP1 does not have these effects. Increasing LPP1 expression in the cancer cells does not affect LPA levels in both tumors and the plasma, even though the cells have enhanced ecto-activity in vitro [84]. These results emphasize the importance of the intracellular activity of LPP1, because LPP1 does not degrade wls-31 and the PAR1 agonist. In addition, the extent of hydrolysis of optimum (10 μM) extracellular LPA concentrations is not fast enough to attenuate acute response, such as Ca^2+^ mobilization, which occur in 30 s [84].

In our recent report, increasing LPP1 in MDA-MB-231 breast cancer cells decreases the levels of c-Jun and c-Fos in nuclei and suppresses the expression of AP-1 (activator protein 1) -regulated genes including *MMPs* (matrix metalloproteinases) and *CCND1/3* (cyclin D1/D3). This effect can be partially reversed by siRNA against LPP1 [208]. In fact, human breast tumors have significantly higher protein levels of MMP-1, -7, -8, -9, -12, -13, c-Jun, and c-Fos than normal breast tissue [208], which is probably caused by the downregulation of LPP1. *PLPP1* has been recognized as one of 12 genes linked with relapse-free survival in breast cancer patients [225].

The effects of LPP1 and LPP3 in cancers are not always consistent. Nakayama et al. found a biphasic growth pattern of ovarian cancer cells in LPP1 knockout mice [70]. The high level of circulating LPA caused by the decrease in LPP1 facilitates cancer cell growth within the first 2 weeks after inoculation, showing more invasive nodules on the omentum compared with the wild-type mice. However, subsequent tumor growth after 3 weeks is slower in the LPP1 knockout mice than the wild-type mice, leading to formation of smaller tumors [70].

Chatterjee et al. reported a pro-tumorigenic action of LPP3. In their study, the knockdown of LPP3 in U87 and U118 glioblastoma cells inhibits tumor growth in mice, whereas overexpressing LPP3 in SW480 colon cancer cells promotes tumor growth [226]. WM239A melanoma cells failed to degrade extracellular LPA after the knockdown of LPP3, but not LPP1 or LPP2, leading to an impaired chemotaxis toward LPA, which was related to the loss of a self-generated LPA gradient outside cells [227].

LPP2 expression is elevated in transformed cells and a variety of cancer cell lines including MCF7, SK-LMS1, MG63, and U2OS [228]. The upregulation of LPP2 is also shown in many cancers, which is opposite to that for LPP1/3 (Figure 4). The knockdown of LPP2 impairs the anchorage-dependent growth of cancer cell lines and decreases cell proliferation [7,228]. These in vitro data validate LPP2 as a putative cancer target. Our unpublished data indicated that the knockout of LPP2 in breast cancer cells inhibits cell proliferation, but it does not affect migration. The cells with LPP2 knockdown form smaller tumors in mice than the wild-type cells. Different functions of LPPs in cancers are summarized in Figure 3.

The different effects of LPP1/3 and LPP2 on cancers may be reflected by their distinct non-redundant functions. For instance, unlike LPP1 [70] and LPP2 [229] knockout mice, which are viable, LPP3 knockout mice die between E7.5 and 9.5 and fail to form a chorioallantoic placenta and yolk sac vasculature [230]. Wunen and Wunen-2 are *Drosophila* homologues of human LPP [32]. The mutation of Wunens causes the impaired migration and death of primordial germ cells. This can be rescued by human or mouse LPP3, but not human LPP1 or mouse LPP2 [32,231]. The knockdown of LPP2 affects the cell cycle by delaying S-phase entry and cyclin A expression. Conversely, the overexpression of LPP2, but not a catalytically inactive mutant, causes premature S-phase entry, which is accompanied by premature cyclin A accumulation [7]. This effect of LPP2 is not observed with LPP1 and LPP3, where the overexpression of these two isoforms normally inhibits cell growth and migration [84,223]. Divergent subcellular distribution could be another reason. In polarized MDCK cells, LPP1 and LPP3 are differentially located on the apical and basolateral subdomains of the plasma membrane, respectively [40]. Sciorra and Morris found that LPP3, but not LPP1, increases DAG accumulation from PLD-generated PA in HEK293 cells. The authors also showed that PLD and LPP3 co-exist in caveolin-1-enriched detergent-resistant membrane microdomains where PLD and LPP3 act sequentially to generate DAG [38]. This subtle difference in localization may allow LPPs to act on specific intracellular pools of substrates and differentially regulate intracellular signaling. To understand more clearly the effects of LPPs in cancers, investigations need to be expanded to more types of cancer. It is important to understand how the LPP isoforms regulate intracellular signal transduction in addition to the degradations of extracellular LPA and S1P. The intracellular lipid phosphate targets for different LPPs need to be identified. LPP2 is a promising target because it is upregulated in many cancers. Developing LPP2 inhibitors is feasible for cancer therapy.

## 9. Conclusions

Plasma membrane-bound LPPs are responsible for the dephosphorylation of extracellular LPA and S1P through their ecto-activities, and this decreases the activation of the respective cell surface receptors. Intracellular LPPs, which are localized on organelles such as the ER and Golgi, also attenuate post-receptor signaling through various LPA receptors and other GPCRs such as PAR receptors. LPP1–3 have distinct and non-redundant functions in physiological processes beside the common phosphatase activities. In cancers, LPP1/3 are generally downregulated, whereas LPP2 is upregulated. LPP1/3 demonstrate antitumorigenic effects in ovarian and breast cancer cells, but they have opposite effects in melanoma and glioblastoma cells. Emerging evidence suggest that LPP2 may function as a tumor promoter, which is different from LPP1/3. The reason for these differences in LPPs are not completely understood, and they are probably caused by differences in substrate access, localization, and accessibility to intracellular substrates [232], which warrant further investigation. LPPs are promising targets for developing novel approaches to cancer therapy.
